# Supplementary material for: The European tomato market. An approach by export competitiveness maps
Source: PLoS One. 2021 May 3;16(5):e0250867. doi: 10.1371/journal.pone.0250867 (PMC8092757; doi:10.1371/journal.pone.0250867)

S1 Appendix.

S1 Fig. Breakdown of tomato export volumes in the German market.


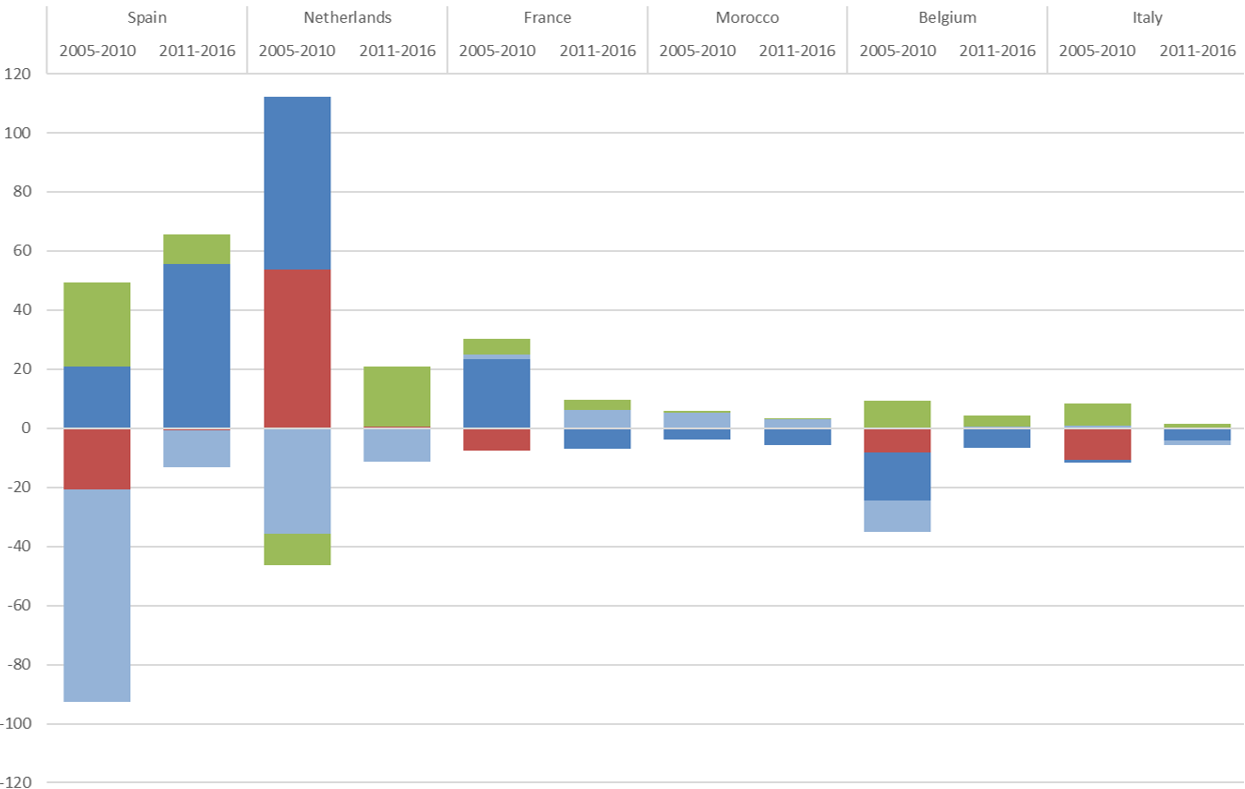


S2 Fig. Breakdown of tomato export volumes in the French market.
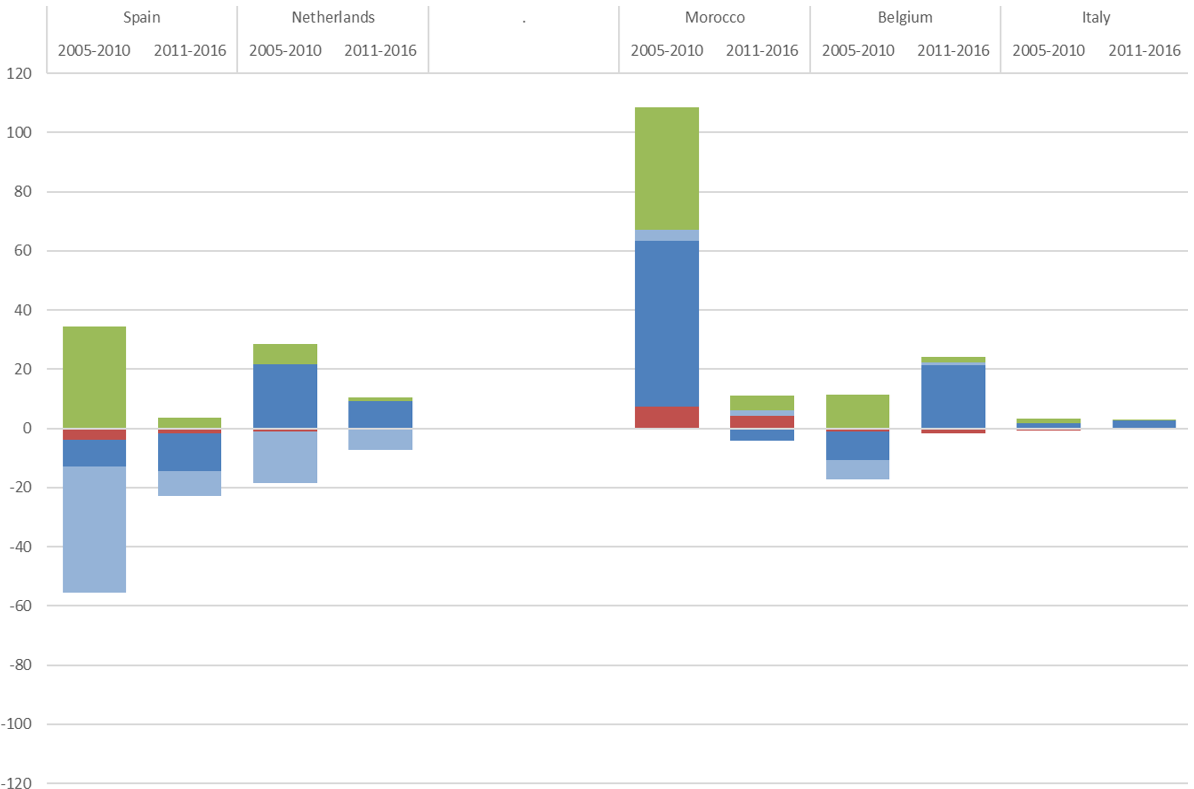


S3 Fig. Breakdown of tomato export volumes in the British market.


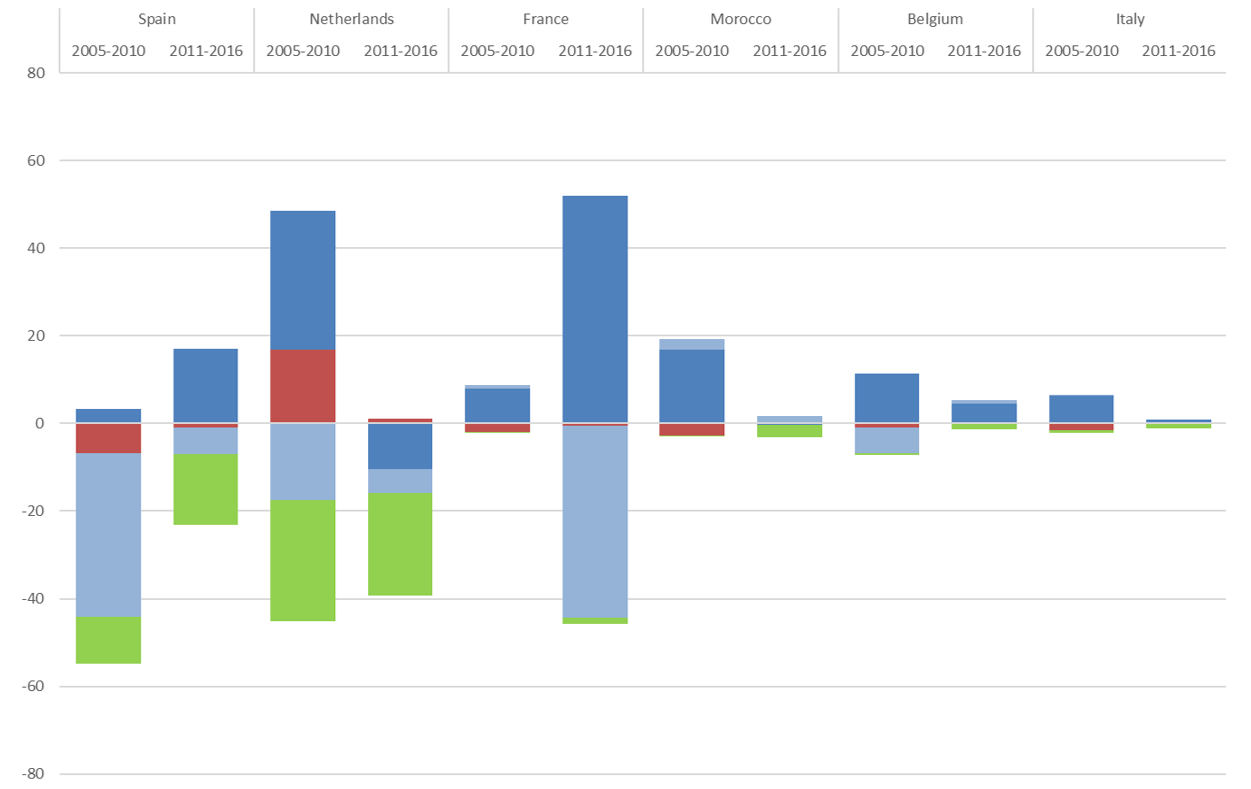

Supplement: S1 Appendix — (DOCX) [file pone.0250867.s001.docx]
