# Supplementary material for: The European tomato market. An approach by export competitiveness maps
Source: PLoS One. 2021 May 3;16(5):e0250867. doi: 10.1371/journal.pone.0250867 (PMC8092757; doi:10.1371/journal.pone.0250867)

S2 APPENDIX.

S1 Fig. Competitiveness map for competing countries in the Dutch market.


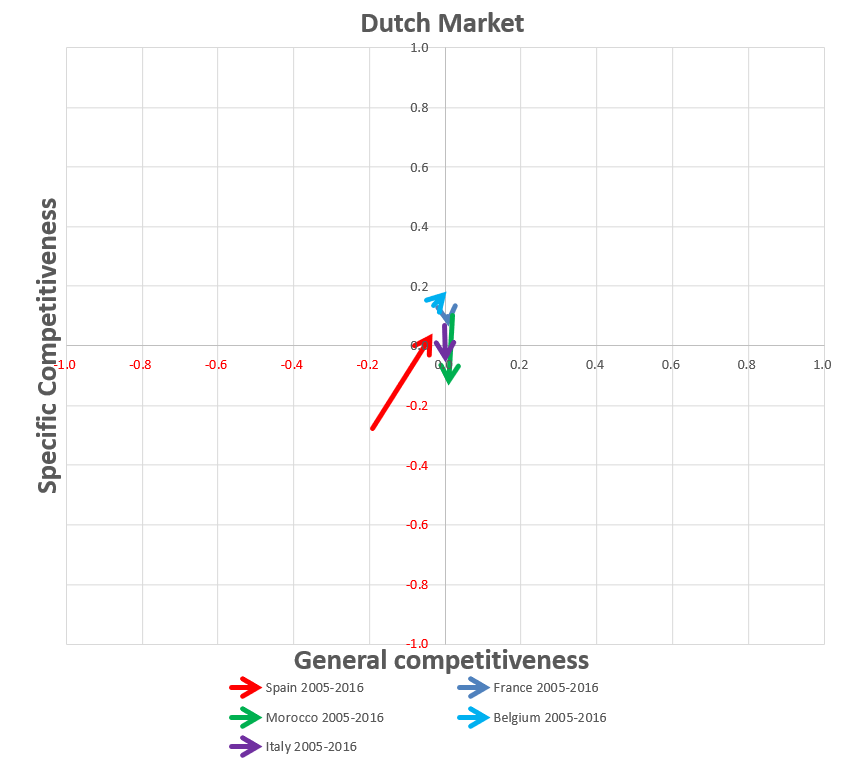


S2 Fig. Competitiveness map for competing countries in the Spanish market.


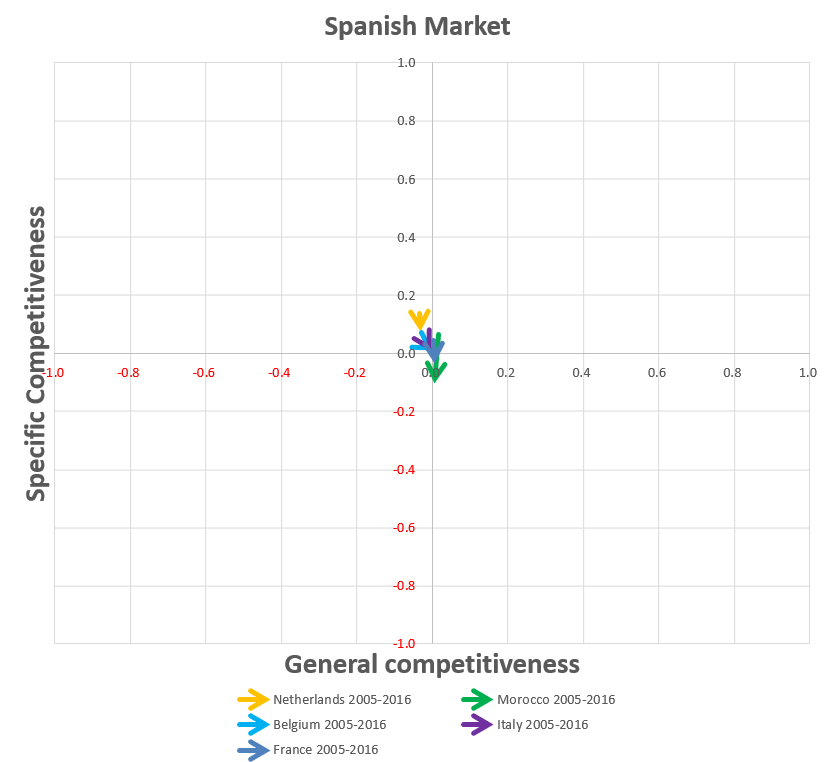


S3 Fig. Competitiveness map for competing countries in the Italian market.


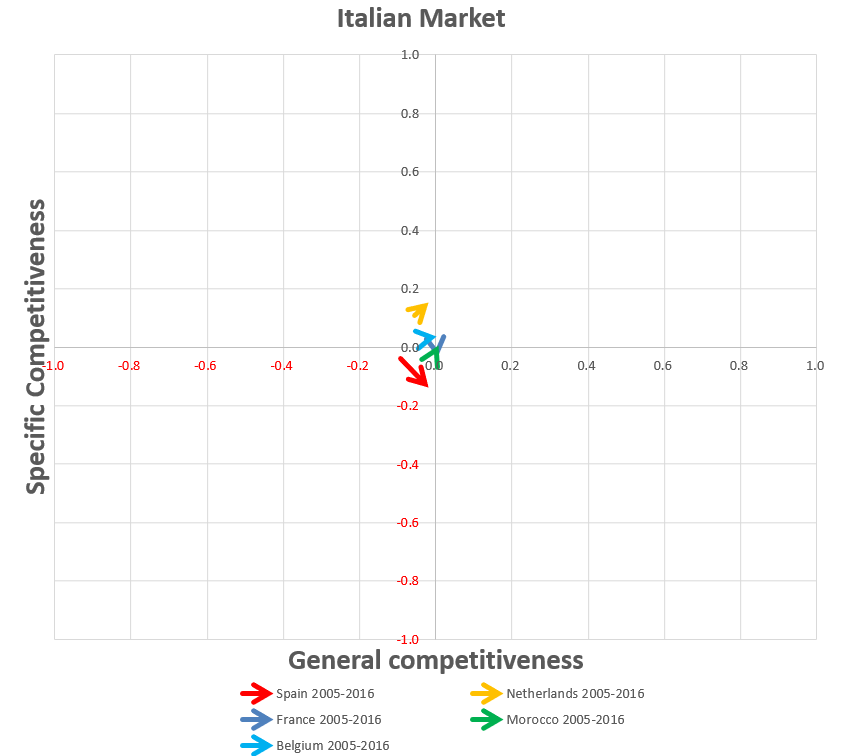


S4 Fig. Competitiveness map for competing countries in the Polish market.


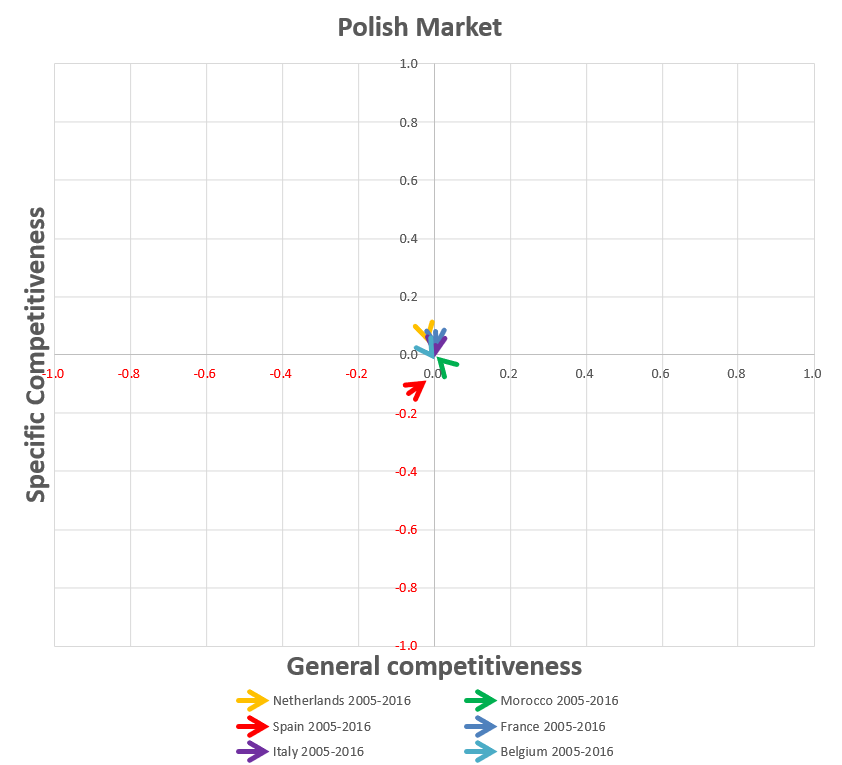


S5 Fig. Competitiveness map for competing countries in the Swedish market.


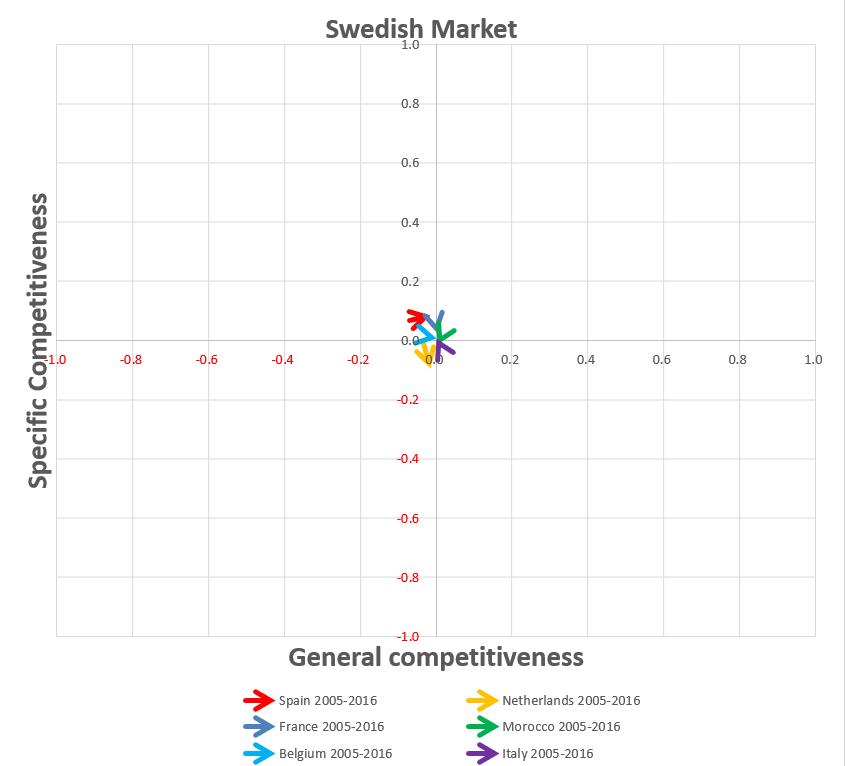


S6 Fig. Competitiveness map for competing countries in the Belgian market.


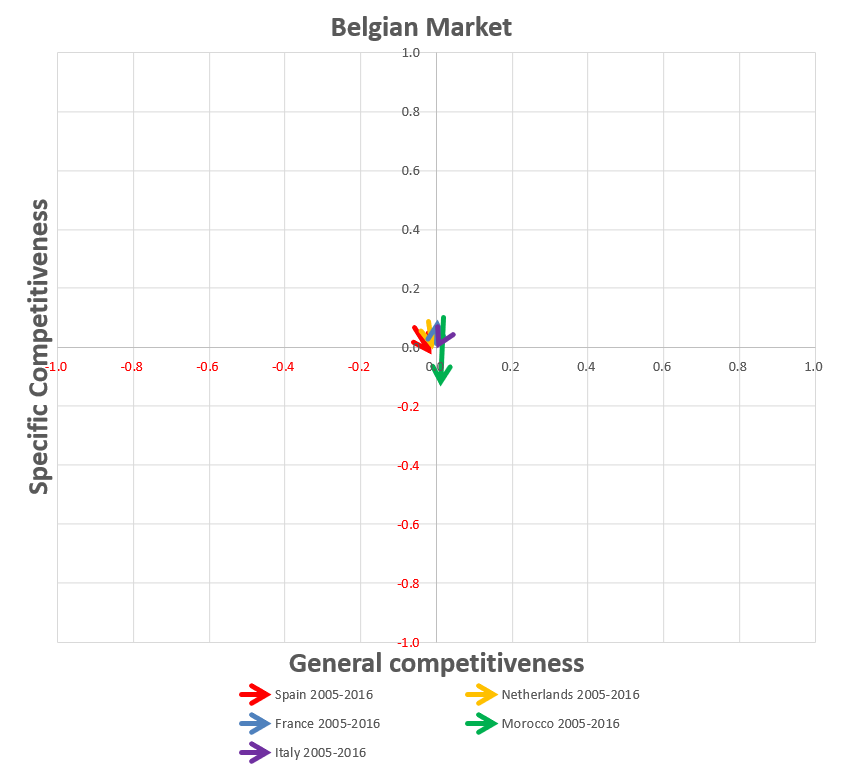

Supplement: S2 Appendix — (DOCX) [file pone.0250867.s002.docx]
