# Supplementary material for: The European tomato market. An approach by export competitiveness maps
Source: PLoS One. 2021 May 3;16(5):e0250867. doi: 10.1371/journal.pone.0250867 (PMC8092757; doi:10.1371/journal.pone.0250867)
Supplement: Attachment — Submitted filename: Response to editor and reviewers.pdf [file pone.0250867.s003.pdf]

Version Review 1

Cover Letter

Mª del Pilar Casado Belmonte
University of Almeria
Carretera Sacramento s/n
04120 La Cañada de San Urbano, Almeria
0034 950 214010
mbelmont@ual.es

Dr. Damien Rousselière
Academic Editor
Plos One

September 30th, 2020

Subject: First revision and resubmission of manuscript PONE-D-20-05952

Dear Dr. Damien Rousselière,

Thank you for your letter and the opportunity to revise our paper. The recommendations offered by the reviewers have been immensely helpful, so we greatly appreciate the insightful comments on revising some aspects of the paper. We fully thank you and the four reviewers the time and effort that you have taken for your comments and suggestions, which we are sure, will greatly enhance our research. We have taken into account the great majority of comments, suggestions and questions of reviewers and have revised our article accordingly.

Immediately after this letter, I have included the reviewer´s comments of the revision and have responded to them individually, indicating exactly how we addressed each concern or problem and describing the changes we have made. All the revisions have been approved by all four authors and I have again been chosen as the corresponding author.

We hope the revised manuscript will better suit *Plos One* , nevertheless should you require any further revisions, we would be grateful to respond.

Sincerely,

María del Pilar Casado-Belmonte
University of Almería, Economics and Business Department

Reviewers Comments, Authors Responses and Manuscript Changes

REVIEWER 1

Thank you for reviewing our manuscript. We greatly appreciate the time and effort that was put into this review as well as the very constructive feedback you have provided that, in our opinion, has enriched our manuscript and has improved the paper's arguments and clarity. We also fully thank you for your positive comments. Please find attached a point-by-point response to your concerns.

COMMENT 1: The text desperately needs a revision of the English level by a native speaker.

RESPONSE: Thanks for your comment. We have hired the services of a professional translator and native English speaker to proofread the text.

COMMENT 2: The title of the paper claims that it will present the likelihood of Spain regaining the position of market leader will be assessed. However, no 'measure' of likelihood is presented. The paper merely concludes that it is likely to happen, in a binary manner. More could be expected for an academic paper. The use of scenarios looking forward should have been considered.

RESPONSE: Thanks for your suggestion. The tittle has been changed so as to show the aim of the study.

CHANGES IN THE TEXT:

Title

The European Tomato Market. An Approach by Export Competitiveness Trending Maps.

COMMENT 3: The literature review lacks references to indexed peer-reviewed journals. The main papers quoted as the basis for the authors (e.g. Molina and Taiariol) are not from indexed peer reviewed journals.

RESPONSE: Thanks for your suggestion. New references have been added. Regarding the reference of Molina and Taiariol (2016), we state that is the only work on export competitiveness using both perspectives (supply and demand), combining the competitors group analysis in the different customer markets comprising the target market. We see the value of the reference even being in a non-indexed journal.

COMMENT 4: The number of figures, graphs and tables is too high. Only information that is directly referred in the main text should be included.

RESPONSE: We appreciate your comment, but the value added of the manuscript is the visualization by maps. Although, we have heeded your advice and some bar charts have been deleted.

COMMENT 5: Chapter 4 fails to properly explain the reasons behind the estimated figures of general and specific competitiveness factors. The section looks more like a pure accounting

exercise. Some loose references are made to factors such as levels of productivity or regulation changes, but patterns, modelling and generalization of the reasons behind are not presented in a clear and structured manner.

RESPONSE: Our aims are to obtain the competitive position between competitor countries in the main European markets and to determine whether there are post-crisis profile changes within the competitor exporting economies. In addition, the competitiveness trending maps offer information regarding future commercial trends for the different competitors in each European market.

We appreciate your comments, but it is far from the aim of the paper. Although we consider your suggestions in the Discussion section.

COMMENT 6: The section on conclusions seems a continuation of the discussion chapter. Conclusions should be shorter and more directed towards the innovative character of the methodology and present the main contributions to the literature.

- The last paragraph of the conclusions should always discuss (i) the main difficulties faced in the research and (ii) suggest avenues for further research.

RESPONSE: We are grateful for this comment. We have proceeded to rewrite the conclusion section.

COMMENT 7: Specific comments: Table 1 is rich in information. It deserves further information in the preceding text, particularly the second breakdown level, which is not explained at all. - Idem for Equation 4. Discussion should be made on the reasons that let the authors to choose this normalization method in particular and not other normalization methods.

RESPONSE: Thanks for your comments. Table 1 has been modified in order to improve the explanation. What is more, the normalization process has been rewritten.

COMMENT 8: Minor suggestions: - Lines 51-54. Statements needs to be substantiated with data or reference in the literature. - Line 70. '(…) is the also most widely-cultivated' – English - Figure 1 presents data from 2005. Outdated. - Figures are repeated in the annex (see Figure 1)

RESPONSE: Thank you for your comments. We have considered your minor suggestions. Sorry for the inconvenience.

REVIEWER 2

Thank you for reviewing our manuscript. We greatly appreciate the time and effort that was put into this review as well as the very constructive feedback you have provided that, in our opinion, has enriched our manuscript and has improved the paper's arguments and clarity. We also fully thank you for your positive comments. Please find attached a point-by-point response to your concerns.

COMMENT 1: The manuscript is not presented in an intelligible fashion and written in standard english.

RESPONSE: We thank your comment and apologise for the inconvenience. We have proceeded to have the text reviewed by a professional native English translator and proof-reader.

COMMENT 2: The introduction is not clear in terms of research questions and objectives. readers do not understand which are the main scopes of the articles and there is not a clear research question expressed.

RESPONSE: We have modified the introduction section and the aims have been rewritten.

CHANGES IN THE TEXT:

> *Introduction Section*
>
> *……*
>
> *The objectives of this article are to obtain the competitive position between competitor countries in the main European markets and to determine whether there are post- crisis profile changes in the competitor exporting economies. In addition, the competitiveness trending maps offer information regarding future commercial trends for the various competitors in each European market.*
>
> *…..*
>
> *Thus, this study contributes to the literature in several ways. First, the novelty of the employment of CMS analysis on the impact on each individual European markets. Second, from a methodological perspective, the introduction of competitiveness trending maps provides information about the profile categorization of exporting economies and illustrates graphically the post crisis profile changes on the European tomato market.*

COMMENT 3: The focus county is Spain but why? in other words, why Spain is interesting in the international scenario of tomatoes market? this aspect is not clear.

RESPONSE: We thank your comment. We see your point and we have changed the focus in the new version of the manuscript. Now the focus is the European market and not only Spain.

REVIEWER 3

Thank you for reviewing our manuscript. We greatly appreciate the time and effort that was put into this review as well as the very constructive feedback you have provided that, in our opinion, has enriched our manuscript and has improved the paper's arguments and clarity. We also fully thank you for your positive comments. Please find attached a point-by-point response to your concerns.

COMMENT 1: The methodology and the graphical solutions hypothesised are interesting, as well as the descriptive picture derived from indicators. However: the use of only quantities traded does not provide enough evidence for the Result and Conclusions sections. CMS is a descriptive method. Its enlargement to demand factors weakens the approach if there is no room for quality related sources of competitive advantage to be ascertained. The role of price, at least, in the different importing countries should be explicitly dealt with. Also other relevant dimensions, such as logistic, SPS standards etc. should be considered and documented. An alternative could be to go back to supply only, integrate the numbers with information on the different players and have a simplified and sounder picture and discussion.

RESPONSE: We agree with reviewer's comment and see his/her point. We have included in the Discussion section the role of strategies based on cost/price and differentiation in the main competitors from the supply side. In addition, the Introduction section has been reinforced with price information.

CHANGES IN THE TEXT:

Introduction Section

Figure 10. Evolution of the tomato production of export competitors in the European market [mt] (2005-2016).

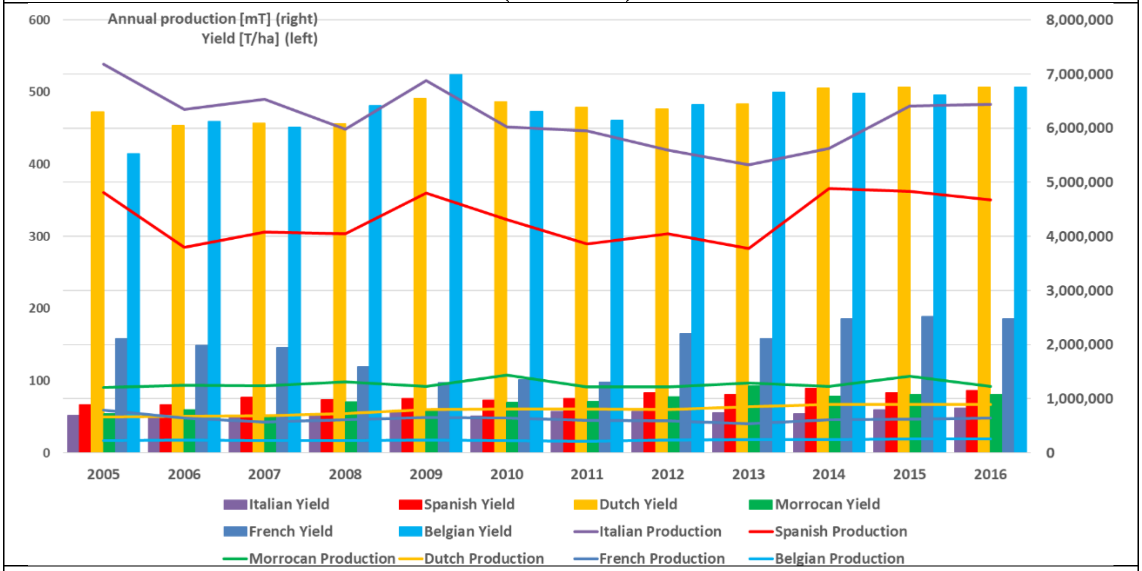

Source: Own elaboration with data from COMTRADE (2018).

In terms of the volume of production in the European Union, Italy is the largest European producer, although the area cultivated is decreasing (Figure 10) and the yield obtained was the lowest among European exporting countries in 2016 (61.94 [Tn/ha]). Spain stands out as

the second largest producer, with a maximum production of 4,888 thousand tonnes in 2014. Like Italy, the area harvested has decreased over the period under consideration and though it has a slightly higher yield than Italy (80.80 [Tn/ha]). Spanish exports are concentrated in the months from October to May (Figure 9) and the average price is of 1.17 [USD/Kg]. The Netherlands does not stand out among the largest European producers, but shows a slight upward trend in its production, due to both an increase in the area harvested and its yield (Capobianco et al, 2020), presenting the highest yield in European production together with Belgium, with 506.90 [Tn/ha] in 2016. The Netherlands is also the world's second largest exporter, due to the re-export of products which are not produced in the Netherlands. Dutch exports are concentrated in the months April to October (Figure 9) and the average price is of 1.59 [USD/kg]. France and Belgium show the least significant values in European production. In both countries, the area harvested has decreased, while yield has increased. The average yield in France is 186.10 [t/ha] and the average price of French and Belgian exports are around 1.41 [dollar/kg] and 1.29 [dollar/kg] respectively. Finally, Morocco's production has increased slightly, mainly due to a progressive increase in its yield (80.80 [Tn/ha]), which is similar to the Spanish yield. The average price of Moroccan exports fluctuates around 1.14 [dollars/kg].

Discussion Section

….

In this sense, trade policies basically consider two types of competitiveness strategies, via costs or product differentiation (Porter, 1990). The strategy via costs enables competitiveness through selling in bulk and offering the products at low prices in the market. From the export price data provided in the introduction section, it can be seen that Spain adopted the costs strategy in the pre-crisis period, but simultaneously was losing competitiveness against the Netherlands (Figures 19, 21, 23, 25, 26 - 30) and finally lost the export leadership in 2009 to the Netherlands. After 2012, when Morocco started to export through a new Free Trade Agreement to the European Union, Spain had to change its strategy, focusing on product differentiation, since it cannot compete with Morocco regarding costs. In this respect, Morocco has a more competitive production cost structure with lower labour costs (1.12 euros/hour) than Spain (8.37 euros/hour) (Cáceres-Hernadez et al., 2018). It is worth noting that the Netherlands stands out in the post-crisis period as the leading country, with its strategy of purchasing in bulk, re-exporting and splitting up the product presentation, which has brought about an increase in exports, even without having its own production of tomatoes for internal supply and maintenance of its client markets (Figure 10).

…..

COMMENT 2: The statistical analysis is probably performed appropriately and rigorously. However, both limitations in the implementation of Plos Data policy (see point 3 below) and a seriously lacking presentation of tables and graphs (see point 4 below) does not allow to be sure of that.

RESPONSE: We thank your comment. We confirm that the methodology has been conducted in a rigorous and appropriate way. What is more, the data in excel is uploaded. We will provide the format of tables and figures in the format required. The adoption of the required format is not a problem for the authors.

COMMENT 3: Original Comtrade data used for the indicators are not provided in the annexes to the manuscript.

RESPONSE: We attach the excel file.

COMMENT 4: The manuscript is written in standard English. However: unit measures in some tables are missing or wrong. Title and labels of tables are often missing, for both table 1 in the text and the other tables.

RESPONSE: Thank you for your comment. The mistakes detected have been corrected.

COMMENT 5: the definition of variables (p. 10-11) is unclear, the disctinction between world flows and target country's flows is mainly a matter of intuition. This also makes unclear the meaning of the adopted breakdowns.

RESPONSE: Thank you for your suggestion. We have changed Table 1 and the new figures show in a clearer way the information.

CHANGE IN THE TEXT:

2.4. Constant market share sub-section

Table 1. Interpretation of different factors in the first and second level of exports decomposition of a region, country or commercial area makes in a target market.

<table>
<tr><td rowspan="5">First breakdown level</td><td rowspan="5">$\Delta q = S_{j0}\Delta Q_j$
$+ Q_{j0}\Delta S_j$
$+ \Delta Q_j \Delta S_j$
(Equation 1)</td><td>$S_{j0}\Delta Q_j$
Structural Effect</td><td>Expected change in the volume of expected exports (ΔQ) supposing that the worldwide market share, and in the target market of the country under analysis, remain constant (S), is the growth in exports exclusively driven by a growth in demand.</td></tr>
<tr><td colspan="2">(+/-) signs indicate that the growth in demand for this product positively/negatively affects the growth in exports. In other words, if (+) is the growth in demand for the product, j positively affects the growth in exports.</td></tr>
<tr><td>$Q_{j0}\Delta S_j$
Competitiveness Effect</td><td>Difference between real and expected exports and associated with changes in competitivity, in other words, it is the growth in exports through displacement of competitors from the target market, interpreted as a relative improvement in the competitive profile.</td></tr>
<tr><td colspan="2">(+/-) signs indicate that the country is gaining/ losing competitiveness.</td></tr>
<tr><td>$\Delta Q_j \, \Delta S_j$
Interaction Effect</td><td>Measures the influence of interaction between changes in market share with changes in demand,</td></tr>
</table>

<table>
<tr><td rowspan="2"></td><td rowspan="2"></td><td></td><td>in other words, the adaptation of market shares to the growth in imports of the markets supplied.</td></tr>
<tr><td colspan="2">(–) sign indicates that exporters have lost market share in dynamic markets and gained market share in less dynamic markets.</td></tr>
<tr><td rowspan="11">Second breakdown level</td><td rowspan="11">$\Delta q = S_{T0}\Delta Q_j$
$+(S_{j0}\Delta Q_j - S_{T0}\Delta Q_j)$
$+Q_{j0}\,\Delta S_T$
$+(Q_{j0}\,\Delta S_j - Q_{j0}\,\Delta S_T)$
$+\left(\frac{Q_{T1}}{Q_{T0}} - 1\right) Q_{j0}\Delta S_j$
$+\Delta Q_j\,\Delta S_j - \left[\left(\frac{Q_{T1}}{Q_{T0}} - 1\right) Q_{j0}\Delta S_j\right]$

(Equation 2)</td><td>$S_{T0}\Delta Q_j$
Growth Effect</td><td>Expected change in export volumes (ΔQ) supposing that the worldwide market share remains constant, it is the export growth attributed to demand for the product j.</td></tr>
<tr><td colspan="2">(+) sign indicates an increase in demand for the product analysed on a worldwide level.</td></tr>
<tr><td>$S_{j0}\Delta Q_j - S_{T0}\Delta Q_j$
Market Effect</td><td>Additional change expected in export volume (ΔQ) supposing that market share remains constant in the target market, is the growth in exports driven exclusively by a growth in demand for the product j in the target market.</td></tr>
<tr><td colspan="2">(+) sign indicates that the exporting country tends to concentrate its exports to markets that are growing rapidly.</td></tr>
<tr><td>$Q_{j0}\,\Delta S_T$
General Competitiveness Effect</td><td>Expected change in exports attributable to changes in competitiveness in general on a global level.</td></tr>
<tr><td colspan="2">(+/-) indicate that the country is gaining / losing competitiveness at global level.</td></tr>
<tr><td>$(Q_{j0}\,\Delta S_j - Q_{j0}\,\Delta S_T)$
Specific Competitiveness Effect</td><td>Expected change in exports attributable to changes in the specific competitiveness of the target market.</td></tr>
<tr><td colspan="2">(+/-) indicate that the country is gaining / losing competitiveness at specific market.</td></tr>
<tr><td>$\left(\frac{Q_{T1}}{Q_{T0}} - 1\right) Q_{j0}\,\Delta S_j$
Pure interaction Effect</td><td>Measures the interaction between changes in the share of an exporter in the target market and changes in demand on a worldwide level.</td></tr>
<tr><td colspan="2"></td></tr>
<tr><td>$\Delta S_j\Delta Q_j - \left[\left(\frac{Q_{T1}}{Q_{T0}} - 1\right) Q_{j0}\,\Delta S_j\right]$
Residual interaction Effect</td><td>Measures the interaction between changes in the share of an exporter in the target market and</td></tr>
</table>

			changes in the level of demand in the target market.

Whereas:

Δq Breakdown of the analysed competitor country's export volume

S_{j0} Analysed competitor country's market share S in the target market j (at moment t_0)

ΔS_j Absolute change rate Δ of analysed competitor country's market share S in the target market j (between t_1-t_0)

S_{T0} Analysed competitor country's market share S in the global market T (at moment t_0)

ΔS_T Absolute change rate Δ of analysed competitor country's market share S in the global market T (between t_1-t_0)

Q_{j0} Competing countries group's export volume Q of in the target market j (at moment t_0)

ΔQ_j Absolute change rate Δ of the competing countries group's export volume Q in the target market j (between t_1-t_0)

Q_{T0} Competing countries group's export volume Q of in the global market T (at moment t_0)

ΔQ_T Absolute change rate Δ of the competing countries group's export volume Q in the global market T (between t_1-t_0)

$\left(\frac{Q_{T1}}{Q_{T0}} - 1\right)$ Relative change rate of the competing countries group's export volume Q in the global market T (between t_1-t_0)

COMMENT 6: Label and colors in the map are confusing. To be checked and revised making them more consistent throughout the maps

REPONSE: We thank your advice. All labels and colours have been improved.

COMMENT 7: Pages from 12 to beginning 14 are repetitious and can be synthesized

RESPONSE: Thank you for your suggestion. The bar figures have been modified and some of them have been deleted.

COMMENT 6: Some editing work throughout the text is recommended

RESPONSE: We have proceeded to review the text. Thank you.

<u>REVIEWER 4</u>

Thank you for reviewing our manuscript. We greatly appreciate the time and effort that was put into this review as well as the very constructive feedback you have provided that, in our opinion, has enriched our manuscript and has improved the paper's arguments and clarity. We also fully thank you for your positive comments. Please find attached a point-by-point response to your concerns.

COMMENT 1: General comments. This article deals with the analysis of tomato export competitiveness. The article focuses on Spain position according to its main competitors. The methodology used is the Constant Market Share (CMS) approach. The results are presented in a new graphical way using trending maps. The article highlights important results and develops an interesting discussion. Nevertheless I would suggest some improvements before publication. The improvements I suggest are threefold.

RESPONSE: Thank you for your positive comment.

COMMENT 2: First, I am not sure the methodology of the CMS is properly presented. The current presentation in table 1 is quite difficult to follow. The variables used in the equations are poorly explained and the indices (j or j0, T0….) are not explained. At the end, I was lost! And this is very important that the reader to fully understand the methodology developed.

RESPONSE: Thank you for the comment. We have proceeded to modify Table 1 and to add an explanation of variables at the end of Table 1. We consider that the changes have helped to gain clarity to the methodology of CMS. Again, thanks for your comment.

CHANGE IN THE TEXT:

2.4. Constant market share sub-section

Table 1. Interpretation of different factors in the first and second level of exports decomposition of a region, country or commercial area makes in a target market.

<table>
<tr>
<td rowspan="3">First breakdown level</td>
<td rowspan="3">$\Delta q = S_{j0}\Delta Q_j$
$+ Q_{j0}\Delta S_j$
$+ \Delta Q_j \Delta S_j$
(Equation 1)</td>
<td>$S_{j0}\Delta Q_j$
Structural Effect</td>
<td>Expected change in the volume of expected exports (ΔQ) supposing that the worldwide market share, and in the target market of the country under analysis, remain constant (S), is the growth in exports exclusively driven by a growth in demand.</td>
</tr>
<tr>
<td colspan="2">(+/-) signs indicate that the growth in demand for this product positively/negatively affects the growth in exports. In other words, if (+) is the growth in demand for the product, j positively affects the growth in exports.</td>
</tr>
<tr>
<td>$Q_{j0}\Delta S_j$
Competitiveness Effect</td>
<td>Difference between real and expected exports and associated with changes in competitivity, in other words, it is the growth in exports through</td>
</tr>
</table>

<table>
<tr><td rowspan="4"></td><td rowspan="4"></td><td></td><td>displacement of competitors from the target market, interpreted as a relative improvement in the competitive profile.</td></tr>
<tr><td colspan="2">(+/-) signs indicate that the country is gaining/ losing competitiveness.</td></tr>
<tr><td>$\Delta Q_j \ \Delta S_j$
Interaction Effect</td><td>Measures the influence of interaction between changes in market share with changes in demand, in other words, the adaptation of market shares to the growth in imports of the markets supplied.</td></tr>
<tr><td colspan="2">(–) sign indicates that exporters have lost market share in dynamic markets and gained market share in less dynamic markets.</td></tr>
<tr><td rowspan="8">Second breakdown level</td><td rowspan="8">$\Delta q = S_{T0}\Delta Q_j$
$+(S_{j0}\Delta Q_j - S_{T0}\Delta Q_j)$
$+Q_{j0} \ \Delta S_T$
$+(Q_{j0} \ \Delta S_j - Q_{j0} \ \Delta S_T)$
$+\left(\frac{Q_{T1}}{Q_{T0}} - 1\right) Q_{j0}\Delta S_j$
$+\Delta Q_j \ \Delta S_j - \left[\left(\frac{Q_{T1}}{Q_{T0}} - 1\right) Q_{j0}\Delta S_j\right]$

(Equation 2)</td><td>$S_{T0}\Delta Q_j$
Growth Effect</td><td>Expected change in export volumes (ΔQ) supposing that the worldwide market share remains constant, it is the export growth attributed to demand for the product j.</td></tr>
<tr><td colspan="2">(+) sign indicates an increase in demand for the product analysed on a worldwide level.</td></tr>
<tr><td>$S_{j0}\Delta Q_j - S_{T0}\Delta Q_j$
Market Effect</td><td>Additional change expected in export volume (ΔQ) supposing that market share remains constant in the target market, is the growth in exports driven exclusively by a growth in demand for the product j in the target market.</td></tr>
<tr><td colspan="2">(+) sign indicates that the exporting country tends to concentrate its exports to markets that are growing rapidly.</td></tr>
<tr><td>$Q_{j0} \ \Delta S_T$
General Competitiveness Effect</td><td>Expected change in exports attributable to changes in competitiveness in general on a global level.</td></tr>
<tr><td colspan="2">(+/-) indicate that the country is gaining / losing competitiveness at global level.</td></tr>
<tr><td>$(Q_{j0} \ \Delta S_j - Q_{j0} \ \Delta S_T)$
Specific Competitiveness Effect</td><td>Expected change in exports attributable to changes in the specific competitiveness of the target market.</td></tr>
<tr><td colspan="2">(+/-) indicate that the country is gaining / losing competitiveness at specific market.</td></tr>
</table>

		$\left(\frac{Q_{T1}}{Q_{T0}}-1\right)Q_{j0}\,\Delta S_j$<br>Pure interaction Effect	Measures the interaction between changes in the share of an exporter in the target market and changes in demand on a worldwide level.
		$\Delta S_j \Delta Q_j - \left[\left(\frac{Q_{T1}}{Q_{T0}}-1\right)Q_{j0}\,\Delta S_j\right]$<br>Residual interaction Effect	Measures the interaction between changes in the share of an exporter in the target market and changes in the level of demand in the target market.

Whereas:

Δq Breakdown of the analysed competitor country's export volume

S_{j0} Analysed competitor country's market share S in the target market j (at moment t_0)

ΔS_j Absolute change rate Δ of analysed competitor country's market share S in the target market j (between t_1-t_0)

S_{T0} Analysed competitor country's market share S in the global market T (at moment t_0)

ΔS_T Absolute change rate Δ of analysed competitor country's market share S in the global market T (between t_1-t_0)

Q_{j0} Competing countries group's export volume Q of in the target market j (at moment t_0)

ΔQ_j Absolute change rate Δ of the competing countries group's export volume Q in the target market j (between t_1-t_0)

Q_{T0} Competing countries group's export volume Q of in the global market T (at moment t_0)

ΔQ_T Absolute change rate Δ of the competing countries group's export volume Q in the global market T (between t_1-t_0)

$\left(\frac{Q_{T1}}{Q_{T0}}-1\right)$ Relative change rate of the competing countries group's export volume Q in the global market T (between t_1-t_0)

COMMENT 3: Second, I find the mapping of the results very interesting; and I agree, this is much easier for the reader to understand the results. The analysis of the results gives an overview of the evolution of the export competitiveness in the tomato market for different countries. But I am not convinced that the analysis of the maps can give any idea of what will happen in the next period. The author(s) mention that, based on the results, we can 'foresee" some evolutions. I am not convinced. To do so, I would have expected an analysis of the underlying factors explaining the trends at play. For instance the author(s) discuss about the role of cherry tomato. I appreciate this discussion. But I would expect a deeper discussion on the determinants of the potential evolution of the productions in the different competitor countries. Such a discussion would have been helpful, especially in the case of Spain (as it is the country under interest). The same remarks holds for the different determinants of consumption of "cherry tomato" in the different target countries.

RESPONSE: Thank you for your comment. We have proceeded to change the old figures 21 and 22. Now, they are shown by exporting economies in each European market (figures 20, 22, 24, 26, 27, 28, 29, 30 and 31). In addition, the trending maps have been applied with linear model regression.

With regards to the underlying factors, the discussion has been enriched with the strategy differentiation.

CHANGES IN TEXT:

Discussion Section

…..

In this sense, trade policies basically consider two types of competitiveness strategies, via costs or product differentiation (Porter, 1990). The strategy via costs enables competitiveness through selling in bulk and offering the products at low prices in the market. From the export price data provided in the introduction section, it can be seen that Spain adopted the costs strategy in the pre-crisis period, but simultaneously was losing competitiveness against the Netherlands (Figures 19, 21, 23, 25, 26 - 30) and finally lost the export leadership in 2009 to the Netherlands. After 2012, when Morocco started to export through a new Free Trade Agreement to the European Union, Spain had to change its strategy, focusing on product differentiation, since it cannot compete with Morocco regarding costs. In this respect, Morocco has a more competitive production cost structure with lower labour costs (1.12 euros/hour) than Spain (8.37 euros/hour) (Cáceres-Hernadez et al., 2018). It is worth noting that the Netherlands stands out in the post-crisis period as the leading country, with its strategy of purchasing in bulk, re-exporting and splitting up the product presentation, which has brought about an increase in exports, even without having its own production of tomatoes for internal supply and maintenance of its client markets (Figure 10).

The results offered by CMS methodology has enabled the display of results through export competitiveness trending maps along with traditional numerical tables and bar graphs in order to facilitate the interpretation of the components of competitiveness for competitors in each of its main European markets. Moreover, with the discriminated analysis of only four of the most important European markets, more than half of the intra-community market is covered (62.27%). The importance of the tomato both as the vegetable with the greatest presence in international trade and the European market as the leading importer/exporter on a worldwide level of this vegetable, underline the relevance of undertaking a tomato export competitiveness study in the intracommunity trade context, in order to analyse the possibilities of Spain being able to recover the leadership lost to the Netherlands in 2009.

….. In such a way, another topic that deserves attention in the context of international trade is the role of the private food standards and certifications, which are becoming increasingly important. In the European context, Global GAP certification is deemed as one of the most important and preferable for retailers (Coel et, al, 2012; Henson et al., 2011). The compliance with Global GAP standards involves costs that can be barriers to countries following a costs strategy (Fiankor, Flachsbarth, Masood, & Brümmer, 2017). In the European context, Morocco is the competitor with a strategy based on costs. Accordingly, it would be interesting to analyse the percentage of export production of tomatoes that is certified and required by the French market since France is the main market for Morocco.

COMMENT 4. Third, as the author(s) work on the tomato product, I was expecting a discussion on the role of private standards and especially GLOBAL GAP. Is there a link between the trend observed and the certification with GLOBAL GAP? At the end, I was very surprised that this important determinant of imports (linked to the requirement of retailers) was not accounted for in the analysis. I think this is a very important element to include in the article.

RESPONSE: We thank your comment and suggestions. We wholly agree with you with the importance of the Global GAP certification as a way to entry in markets. We have mentioned this fact in the discussion section.

CHANGES IN THE TEXT:

Discussion Section

….. In this vein, another topic that deserves attention in the context of international trade is the role of the private food standards and certifications, which are becoming increasingly important. In the European context, Global GAP certification is deemed as one of the most important and preferable for retailers (Coel et, al, 2012; Henson et al., 2011). The compliance with Global GAP standards involves costs that can be barriers to countries following a costs strategy (Fiankor, Flachsbarth, Masood, & Brümmer, 2017). In the European context, Morocco is the competitor with a strategy based on costs. Accordingly, it would be interesting to analyse the percentage of export production of tomatoes that is certified and required by the French market since France is the main market for Morocco.

Reference Section

Colen, L., Maertens, M., and Swinnen, J. (2012). Private standards, trade and poverty: GlobalGAP and horticultural employment in Senegal. The World Economy, 35(8), 1073– 1088.

Fiankor, D. D. D., Flachsbarth, I., Masood, A., & Brümmer, B. (2020). Does GlobalGAP certification promote agrifood exports?. European Review of Agricultural Economics, 47(1), 247-272.

Henson, S., Masakure, O., and Cranfield, J. (2011). Do fresh produce exporters in subSaharan Africa benefit from GlobalGAP certification? World Development, 39(3), 375– 386.

COMMENT 5: The equations in table 1, especially equation 3 should be explained in details.

RESPONSE: Thank you for your comment. We have changed Table 1 and we have made the explanation simpler to avoid causing confusion.

COMMENT 6: Normalization: some details should be given on the normalization procedure. What is the distribution of the indexes before and after the normalization?

RESPONSE: We thank your comment. We have rewritten the explanation of normalization process, including the response curve of normalized data (new Figures 14 and 15) and new figures comparing the data distribution after and before the normalization process (Figures 16 and 17).

CHANGES IN THE TEXT:

2.4.1. Normalization of competitiveness indexes sub-section

The design of multidimensional index systems requires the integration of a wide range of indicators with dissimilar characteristics. Methodological guides to implement index systems are supplied by Economic Commission for Latin America and the Caribbean (CEPAL) but these only provide general guidelines for their construction (Quiroga Martínez, 2009). Despite the lack of instructive guides for the construction of multidimensional systems, an adaptation of the guide for the construction of composite indexes produced by the Organization for Economic Co-operation and Development (OCDE) can be used. The "Handbook on constructing composite indicators" (OCDE, 2008) by the Joint Research Centre-European Commission recommended that normalization of indicators should be carried out to render the variables comparable. The normalization of indexes means adjusting values measured on different scales to a notionally common scale, without distorting differences in the ranges of values. As first step, it is necessary to select suitable normalization procedure that respect both the theoretical framework and the data properties. Then, attention needs to be paid to extreme values and the presence of outliers in the dataset as they may become unintended benchmarks. Subsequently, scale adjustments must be made and highly skewed indicators transformed if necessary. In this case of study a sigmoidal normalization was selected (Equation 3) in order to rank between [+1, -1] the original competitiveness values obtained with the CMS methodology.

$$NCV = \frac{2}{\left(1+e^{(-OCV*SF)}\right)} - 1 \qquad \textit{(Equation 3)}$$

Where, NCV is the final Normalized Competitiveness Value, OCV is the Original Competitiveness Value and the SF is an appropriately selected scalar factor according to the order of the original data (SF= 0.0000001). Sigmoidal normalization was selected as a transformation function since the response curve of the normalized values of general competitiveness (Figure 13) and specific competitiveness (Figure 14) are located mainly in the linear zone, with no values located at the extremes that could generate saturated normalized values. Moreover, new outliers were not generated due to the normalization of the data.

Figure 13. Response curve of normalized data of general competitiveness

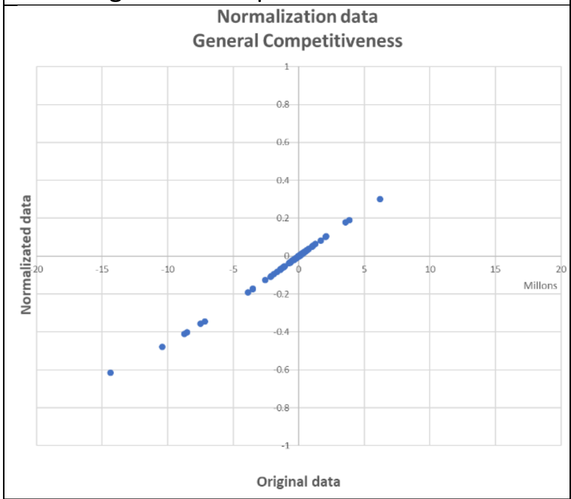

Figure 14. Response curve of normalized data of specific competitiveness

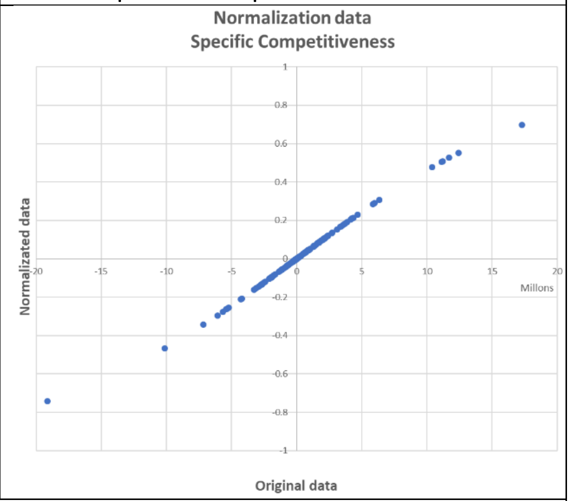

Source: Own elaboration with data from COMTRADE (2018).

Although the main purpose of the process of normalization in a multidimensional index system is to delimit the value rank, the process of normalization has helped to improve some normality characteristics of the data (Figure 15 and Figure 16).

Figure 15. Distribution of the general competitiveness indexes before and after the normalization.

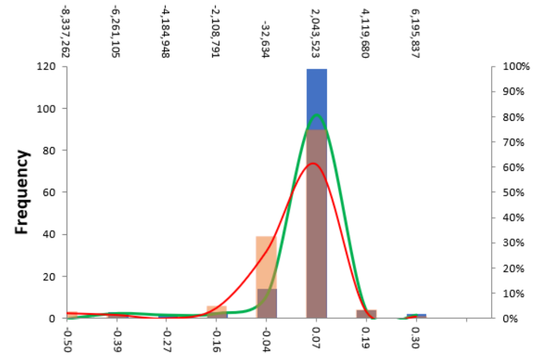

Figure 16. Distribution of the specific competitiveness indexes before and after the normalization.

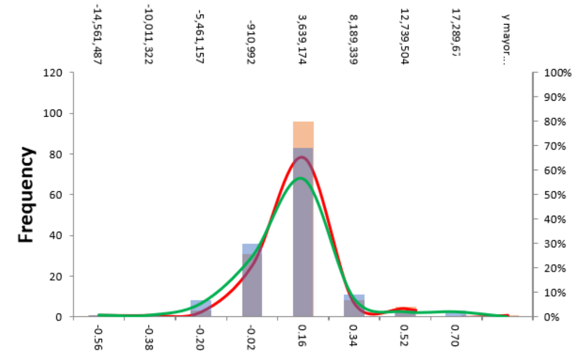

Original data frequency — Original data distribution — Normalized data frequency — Normalized data distribution

Source: Own elaboration with data from COMTRADE (2018).

COMMENT 7: P.12, l. 296, it is mentioned "future commercial trends". I'm not convinced about this. Please develop!

RESPONSE: Thank you for your comment. In order to show the future commercial trends, we have used the linear regression model to make the prediction of the period encompassing (2017-2021) based on COMTRADE database (2005-2016). New figures have been added in the Results Section, namely figures 20, 22, 24, 26, 27, 28, 29, 30 and 31.

CHANGES IN THE TEXT:

2.4.2. Export competitiveness trending maps sub-section

……

In addition, not only does the presentation of the results by competitiveness maps in vectorial show the evolution between periods, but also the commercial future trends obtained by linear model regression in each exporting economy (2017-2021). The ideal export competitiveness situation for an export economy is the location of its vectors in quadrant A, determined by both positive axes, along with a vectoral direction oriented towards the upper right corner.
These innovative competitiveness trending maps improve the interpretation of results and offer complementary information on the evolution of each competitor in the target market through the module, direction and sense of its vector. As can be seen in Figure 17, the Cartesian axes of the 2D space, determine four quadrants coinciding with the different alternatives of general and specific competitiveness that can be adopted by the exporting-supplying countries in the main European tomato markets.

COMMENT 8: P.13 description of group AI, AII, AIII. These groups are not mentioned on figure 12. A clear description should be given (with number or location on the graphic).

RESPONSE: Thank you for the comment. We firmly agree with the reviewer's comment. The new *Figure 18 (old 12). Two-dimensional export competitiveness system for Constant market Share* has been modified with the inclusion in the figure of the AI, AII and AIII groups.

CHANGES IN THE TEXT:

Figure 17. Two-dimensional export competitiveness system for Constant Market Share.

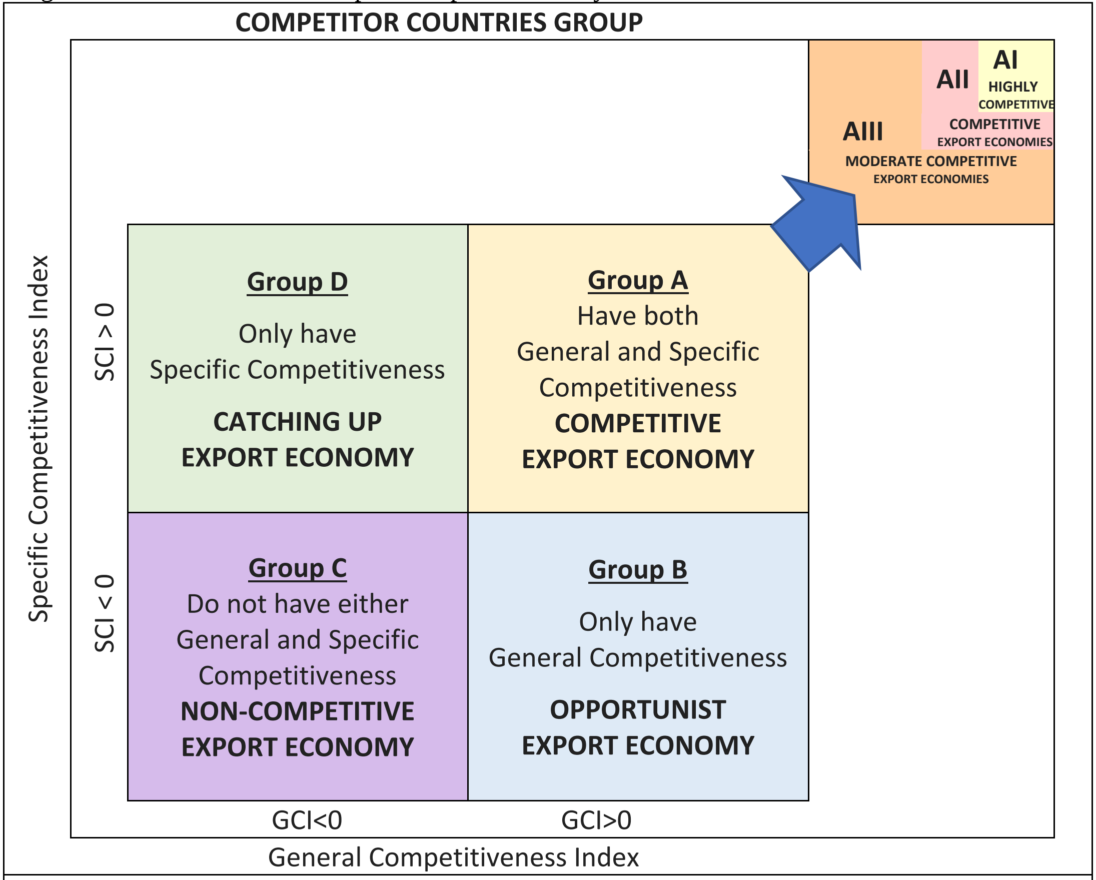

Source: own elaboration based on Widodo (2009).

COMMENT 9: Sometimes confusing. As Spain is the country of interest, may be the discussion should be centered on it. I wonder whether this article could not have a wider scope than mainly Spain. Especially if you succeed in deepening the analysis on the determinants of the highlighted trends.

RESPONSE: We appreciate your comment and we have heeded your advice. We firmly believe that the scope is wider than Spain and we have change the aim of the article so as to adjust to the contribution. We leave the regional view to embrace a more global approach, namely the European market.

CHANGES IN THE TEXT:

> *Title*
>
> *The European Tomato Market. An Approach by Export Competitiveness Trending Maps.*

Introduction Section

The objectives of this article are to obtain the competitive position between competitor countries in the main European markets and to determinate whether there are post- crisis profile changes within the competitor exporting economies. In addition, the competitiveness trending maps offer information regarding future commercial trends for different competitors in each European markets.
