# Supplementary material for: The European tomato market. An approach by export competitiveness maps
Source: PLoS One. 2021 May 3;16(5):e0250867. doi: 10.1371/journal.pone.0250867 (PMC8092757; doi:10.1371/journal.pone.0250867)
Supplement: Attachment — Submitted filename: Response to Reviewers.pdf [file pone.0250867.s005.pdf]

Editor and Reviewers' Comments and Authors Responses

Thank you for reviewing our manuscript. We greatly appreciate the time and effort that was put into this review as well as the very constructive feedback you have provided that, in our opinion, has enriched our manuscript and has improved the paper's arguments and clarity. We also fully thank you for your positive comments. Please find attached a point-by-point response to your concerns.

EDITOR REQUIREMENTS

COMMENT 1: Please address the concerns of reviewer 4 about the "transparency" of your forecastings, as transparency and reproductibility are among the major criteria for publication in PLOS ONE. Explain better the way the predictions are estimated and the limits of your methodology.

RESPONSE: Thank you for your comment. Reviewer 4 asked us to avoid the forecastings of future trends since he/she did not agree with the estimation. He/she suggested that the prediction based on past data was not accurate due to the fact that other aspects, which were not included in past, could affect the direction of future trends, for instance Brexit in the British market.

Accordingly, heeding the advice of Reviewer 4, we have decided to omit the future trend and focus on the discussion of underlying factors that impact on competitiveness in the analysed period.

CHANGES IN THE TEXT:

Title

The European Tomato Market. An Approach by Export Competitiveness Maps

Methodology, Results, Discussion and Conclusion Sections

The text mentioning the future trends has been omitted.

COMMENT 2: Focus the rewriting on the most interesting cases and provide an additional online appendix for the other materials (see comments 3 of referee 4)

RESPONSE: Thank you for your suggestion. We had initially included all the European markets in the main text of the manuscript, namely German, French, British, Dutch, Spanish, Italian, Polish, Swedish and Belgian markets (figures 18-30). Nevertheless, according to the suggestion of Reviewer 4, only the main European markets should be discussed in the main text, that is German, French and British markets.

What is more, in order to avoid the large number of figures in the previous version of the manuscript (30 figures), we have proceeded to include the figures regarding "Breakdown of tomato export volumes in the main markets" (figures 18, 20 and 22) in S1 Appendix. Moreover, the figures regarding "Competitiveness map for competing countries in the (Dutch, Spanish, Italian, Polish, Swedish and Belgian) market" (figures 25 to 30) have been added in the S2 Appendix.

CHANGES IN THE TEXT:

Results Section

Old figures 18, 20 and 22 have been included in S1 Appendix.

Old figure 24 has been omitted.

Old figures 25, 26, 27, 28, 29 and 30 have been changed and included in S2 Appendix.

Old figures 19, 21 and 23 have been changed and renamed as 18, 19 and 20.

COMMENT 3: Follow the advices of reviewer 4 about the abstract and the introduction

RESPONSE: Thank you for your comment. We have proceeded to rewrite the Abstract and the Introduction Section, following the recommendations of Reviewer 4.

CHANGES IN THE TEXT:

Abstract Section

The Abstract section has been reduced and summarised.

Introduction Section

The Introduction Section has been restructured.*Tomato market competitiveness Section*

A new section has been included after the Introduction Section, named "Tomato market competitiveness". In this section, the main aspects of tomato market have been included.

REVIEWER 3

COMMENT 1: This version of the paper has been reviewed substantially. It incorporates all the comments and suggestions made to the original version. I particularly appreciate the clarity in the individualized response to each one of them.

This version substantially improves the clarity and consistency of the general message.

The authors presented in the first version a comprehensive, descriptive, and accounting exercise. In addition, this revised version of the paper introduces a thorough discussion of the reasons behind the changes observed in specific and general competitiveness. This critically adds value to the paper.

Consequently, I recommend the publication of the paper.

RESPONSE: Thank you for your positive and encouraging comments. We are grateful for meeting all your valuable previous comments.

REVIEWER 4

COMMENT 1: The new version of the article accounts for most of my comments on the first version. Table 1 has been improved in a relevant way. A section on GlobalGap has been added. I note that it was mentioned following the French market analysis. It is important to mention also that the UK, as an important fruit and vegetable importer, was the first to impose GlobalGap, mainly through its retailers, which are the main sellers of fruit and vegetable to the British consumers.

RESPONSE: Thank you for your suggestion. We have included in the Discussion Section of the British market, the importance given for this market to voluntary certification. In addition, the Discussion Section has been reinforced with a new paragraph about the role of voluntary quality standards in the Europe context.

CHANGES IN THE TEXT:

Discussion Section

> *In the British market, the third largest in the European market which has recently left the intra-Community market, the Netherlands had the largest market share (Figure 8). In the British market post-crisis, the Netherlands has drastically reduced their specific competitiveness (Figure 20). In addition, Morocco and France were both competitive export economies in pre-crisis, but have lost specific and general competitiveness respectively, turning Morocco into an opportunistic export economy and France into a catching-up export economy. It is worth mentioning the widespread acceptance among British retailers of voluntary quality certifications. In this regard, Global GAP certification is regarded as the first step towards a European harmonised standard for integrated production, rooted in acts such as United Kingdom food safety of 1990 whereby emerged a shift in responsibility [52].*
>
> *……..*
>
> *In addition, the differentiation strategy may be based on quality requirements. In the last years, the agricultural food sector in Europe has shown an increase in the demand for quality safe food to ensure producers to comply with quality and safety requirements [54]. In this regard, mandatory and voluntary quality assurance standards are deemed to pressure producers to meet food safety management systems [52]. Accordingly, Global Gap highlights in Europe with a widespread acceptance. Moreover, in European Union market, it is thought to be a de facto requirement for most major European retailers. In this regard, intra-community competitors have gone steps further to ensure production meets safety and quality requirements in comparison to Morocco.*

References

[52]Alvarez, G. Fair trade and beyond: voluntary standards and sustainable supply chains. In Delivering Performance in Food Supply Chains. 2010: 478-510. Woodhead Publishing.

[54] Gichuki, C. N., Gicheha, S. K., and Kamau, C. W. Do food certification standards guarantee small-sized farming enterprises access to better markets? Effectiveness of marketing contracts in Kenya. International Journal of Social Economics. 2020, 47(4): 445-459.

COMMENT 2: Regarding, the methodology and analysis, a new development appears to deal with the “foreseeing issue” of the paper. Initially the author(s) wrote they could do prediction in the future of competitiveness on the tomato market. I did not agree with this point. To account for this issue, in the new version, the authors mentioned that their methodology shows the evolution between periods, but also the export future trend, obtained by linear regression applied a linear regression model in each economy. This is mentioned in the authors’ answer to my comments. I need more explanations!

For me, this means that the authors only consider that the previous evolution will be the same as the future one. In other words, this evolution will be the relevant one if all factors explaining the previous evolution will stay the same in the future as before. As mentioned by the authors, the Brexit will have an impact in the coming years. Thus, I cannot agree with this way to proceed. In my understanding, the exact determinants of the previous evolutions are not clearly measured, it is difficult to “foresee” something different in trends. I think this is not interesting to add these aspects. I would suggest to the authors to try to explain better the main determinants of the previous evolution.

RESPONSE: Thank you for your comment.

We had calculated the foreseen trend based on past data. In this sense, we wanted to show the direction of the future competitiveness of the countries included in the competitor group. As you mentioned, this method may be accurate under the assumption that other factors are stable as well as constant and, Brexit, for instance, appears as an influential key in coming years.

We see your point that future trend should not be estimated based only on past data and the difficulty of foresing. Thus, we have opted for excluding the trending vector and focusing on the determinants of previous evolution, as you have recommended.

Accordingly, in the competitiveness maps, the discontinuous arrows have been deleted, showing only the pre-crisis and post-crisis positions.

CHANGES IN THE TEXT:

Methodology Section

The way the predictions have been done has been omitted.

Results Section

The figures about competitiveness maps have been modified (new 18, 19 and 20 figures and S2 Appendix), showing only the pre-crisis and post-crisis evolution and excluding future trends.

COMMENT 3: The current version addresses a wider scope than the previous version. I think it is a relevant point. However, this leads to a too long article, with too many Figures

(30 figures!!!). I would suggest to provide an online appendix for instance, with all figures available. The figures proposed in the article should focus on one or two detailed cases, the most interesting cases. The discussion should be based on all the figures available in the appendix. This would lead to a more straightforward reading. The current version gives lots of information, may be too much and it loses the reader. May be some key component of competitiveness should be the main component of the paper. For instance the discussion on cost competitiveness and the illustration with the relative position of Spain and Morocco is very interesting. It should be deepen and only the corresponding graphs should be included in the main text of the article. The same holds for the discussion on private standards.

RESPONSE: Thank you for your suggestions. We wholly agree with you, the inclusion of 30 figures makes the reading difficult to follow. Thus, we have heeded your advice and we have focused on the main European markets, namely German, French and British markets. In this regard, figures regarding other European markets have been included in S2 Appendix. In addition, the figures regarding Breakdown of tomato export volumes in the main markets have been included in S1 Appendix.

Similarly, we have focused the discussion on the behaviour of competitor group in each of the main European markets, highlighting the key aspects of competitiveness. In this regard, we have compared the position of Spain and Morocco and deepened the importance of quality requirements in European context, mainly in British market.

CHANGES IN THE TEXT:

Results Section

Old figures 18, 20 and 22 have been included in S1 Appendix.

Old figure 24 has been omitted.

Old figures 25, 26, 27, 28, 29 and 30 have been changed and included in S2 Appendix.

Old figures 19, 21 and 23 have been changed and renamed as 18, 19 and 20.

Table 2 has been changed, omitting the future trends.

Discussion Section

The Discussion Section has been restructured and deepened with the position of Morocco and Spain and the acceptance of voluntary certifications among retailers in Europe.

COMMENT 4:

COMMENT 4.1.: First, the abstract is too long. I suggest to focus on the contribution of the article, mains the CMS analysis and the graphical representation of the results.

RESPONSE: Thank you for your comment. We have changed the abstract and highlighted the contributions of the study, namely CMS analysis and the maps.

CHANGE IN THE TEXT:

Abstract

> *Most empirical studies examining the export competitiveness of a country in a target market are undertaken by focusing on supply, only analysing the group of competing countries. In addition, if the target market to be analysed is extensive, like the European Union, it is generally analysed as a whole. This study presents an evaluation of the tomato export competitiveness, from a differentiated demand perspective, analysing its main customers markets in the context of European Union. The methodological framework is implemented through Constant Market Share to analyze variations in exports, allowing the portion attributable to competitiveness and segregation into general or specific competitiveness to be quantified. The Constant Market Share was adapted to focus on the differentiated demand so as to observe the influence of the worldwide crisis (2007/08) on the European tomato market. This study allows the analysis of profile changes into the competitor exporting economies. As a contribution to the methodology, this study presents a new graphical way of representing the results of Constant Market Share methodology by means of export competitiveness maps in the European tomato market for the group for each main competitor in each European client market. According to our results, Spain and Belgium are candidate countries to be competitive in the main European markets.*

COMMENT 4.2.: The introduction has also to be reorganised in order to gain in efficiency. In the current version, the objectives of the article are presented on the 6th page. It is too late. From the first two pages, the reader expects an analysis on the impact of regional trade agreements on fruit and vegetable trade. It sounds more an “introductory chapter” than an introduction of a scientific paper. The reader needs to know on the first page what are the question, the contributions, and the literature strand in which the article is included. Another example: the data on prices given in the introduction are more relevant in the discussion of the results. It’s not usual to have some references to the introduction to explain some results at the end. I would suggest to be more straightforward in the introduction, and to use some materials later on.

RESPONSE: Thank you for your comment. We wholly agree with you. Accordingly, we have divided the old Introduction section in two sections, Introduction and Tomato Market Competitiveness sections.

The new Introduction section has been rewritten and reduced. The second section, labelled Tomato Market Competitiveness, includes all relevant information of the tomato market regarding competitiveness.

CHANGES IN THE TEXT

Introduction Section

Tomato Market Competitiveness Section

COMMENT 4.3.: At the end of my comments, I still appreciate the 2D mapping. I think it is interesting for the reader to have some graphical representations. This is a good way to have a clear overview of the competitiveness in a sector, with lots of countries concerned. Anyway, I think the article should gain in structuration and deepen the quantitative analysis of underlying factors, especially to foresee the future competitiveness. The discussions on prices, quality and private standards (as GLOBAL GAP) are very interesting and should be the main part of the discussion.

RESPONSE: Thank you for your suggestion, we have rewritten the Discussion section and have included new analysis of the underlying factors of competitiveness.

CHANGES IN THE TEXT:

Discussion Section

> *…….*
>
> *In the British market, the third largest in the European market which has recently left the intra-Community market, the Netherlands had the largest market share (Figure 8). In the British market post-crisis, the Netherlands has drastically reduced their specific competitiveness (Figure 20). In addition, Morocco and France were both competitive export economies in pre-crisis, but have lost specific and general competitiveness respectively, turning Morocco into an opportunistic export economy and France into a catching-up export economy. It is worth mentioning the widespread acceptance among British retailers of voluntary quality certifications. In this regard, Global GAP certification is regarded as the first step towards a European harmonised standard for integrated production, rooted in acts such as United Kingdom food safety of 1990 whereby emerged a shift in responsibility [52].*
>
> *……..*
>
> *In addition, the differentiation strategy may be based on quality requirements. In the last years, the agricultural food sector in Europe has shown an increase in the demand for quality safe food to ensure producers to comply with quality and safety requirements [54]. In this regard, mandatory and voluntary quality assurance standards are deemed to pressure producers to meet food safety management systems [52]. Accordingly, Global Gap highlights in Europe with a widespread acceptance. Moreover, in European Union market, it is thought to be a de facto requirement for most major European retailers. In this regard, intra-community competitors have gone steps further to ensure production meets safety and quality requirements in comparison to Morocco.*
>
> *……..*
>
> *Spain should take advantage of the double opportunity of Netherland's loss of specific competitiveness and France's loss of general competitiveness. In this market, Spain should compete with colour cherry tomatoes for snacking on outside the home and with small pack sizes by unit in the rest of the varietal categories. Finally, in the*

French market, although its main competitor is Morocco, Spain could take advantage of the specific competitiveness loss of Morocco and support differentiation strategy due to the fact that Spain is not competitive in prices in comparison to Morocco.
