# Supplementary material for: The European tomato market. An approach by export competitiveness maps
Source: PLoS One. 2021 May 3;16(5):e0250867. doi: 10.1371/journal.pone.0250867 (PMC8092757; doi:10.1371/journal.pone.0250867)
Supplement: Attachment — Submitted filename: Review Author.docx [file pone.0250867.s004.docx]

**PONE-D-20-05952R1**

**Comments to author**

General assessment:

This version of the paper has been reviewed substantially. It incorporates all the comments and suggestions made to the original version. I particularly appreciate the clarity in the individualized response to each one of them.

This version substantially improves the clarity and consistency of the general message.

The authors presented in the first version a comprehensive, descriptive, and accounting exercise. In addition, this revised version of the paper introduces a thorough discussion of the reasons behind the changes observed in specific and general competitiveness. This critically adds value to the paper.

Consequently, I recommend the publication of the paper.
